# Real-world Evidence from a Retrospective Multicentre Analysis on First-line Therapy for Metastatic Papillary Renal Cell Carcinoma. A GUARDIANS Project

**DOI:** 10.1016/j.euros.2025.06.011

**Published:** 2025-07-12

**Authors:** Thomas Hilser, Jozefina Casuscelli, Can Aydogdu, Stefanie Zschäbitz, Marco Julius Schnabel, Emily Rinderknecht, Angelika Mattigk, Martin Schostak, Anna-Lisa Volk, Philipp Ivanyi, Jonas Wiegmann, Christopher Darr, Luka Flegar, Subhajit Mandal, Katrin Schlack, Daniel Seidl, Analena Handke, Melanie Klee, Tim Nestler, Marc Rehlinghaus, Sameh Hijazi, Axel Heidenreich, Viktor Grünwald, Pia Paffenholz

**Affiliations:** a Department of Medical Oncology, West German Cancer Center, University Hospital Essen and German Cancer Consortium (DKTK), University Duisburg-Essen, Essen, Germany; b Department of Urology, University Hospital, LMU Munich, Munich, Germany; c Department of Medical Oncology, National Center for Tumor Diseases, Heidelberg University Hospital, Heidelberg, Germany; d Department of Urology, Caritas-St. Josef Medical Center, University of Regensburg, Regensburg, Germany; e Department of Urology, University Hospital Ulm, Ulm, Germany; f Department for Urology, Urooncology, Robot-assisted and Focal Treatment, University of Magdeburg, Magdeburg, Germany; g Logicuro, Berlin, Germany; h Department of Hematology, Hemostasis, Oncology, and Stem Cell Transplantation, Claudia-von Schelling Comprehensive Cancer Center, Hannover Medical School, Hannover, Germany; i Department of Urology, University Hospital Essen, University of Duisburg-Essen, Essen, Germany; j Department of Urology, University of Marburg, Marburg, Germany; k Department of Urology, University Hospital Münster, Münster, Germany; l Department of Urology, Diakonie-Klinikum Stuttgart, Stuttgart, Germany; m Department of Urology, Ruhr University Bochum, Marienhospital Herne, Herne, Germany; n Department of Urology, University Hospital Schleswig-Holstein, Lübeck, Germany; o Department of Urology, Federal Armed Forces Hospital Koblenz, Koblenz, Germany; p Department of Urology, Medical Faculty and University Hospital Düsseldorf, Heinrich Heine University Düsseldorf, Düsseldorf, Germany; q Department of Urology, Ibbenbueren Hospital, Ibbenbueren, Germany; r Department of Urology, Uro-Oncology, Robot Assisted and Reconstructive Urologic Surgery, University of Cologne Faculty of Medicine and University Hospital Cologne, Cologne, Germany; s Center for Integrated Oncology (CIO) Köln-Bonn, Cologne, Germany

**Keywords:** Papillary renal cell cancer, Immune checkpoint inhibitor, Tyrosine kinase inhibitor, First-line treatment

## Abstract

**Background and objective:**

Papillary renal cell carcinoma (pRCC) is a rare disease. The optimal treatment of metastatic pRCC is still unclear. We evaluated real-world treatment outcomes of first-line treatment in this cohort in Germany.

**Methods:**

Patients with advanced or metastatic pRCC were eligible. Adverse events (AEs) were reported according to Common Terminology Criteria for Adverse Events version 5.0. The overall response rate was accessed according to the local standard. Progression-free survival (PFS) was calculated from the start of treatment to progression or death. Descriptive statistics and Kaplan-Meier plots were utilised, where appropriate.

**Key findings and limitations:**

In total, 121 suitable patients (77% male) with a median age of 63 yr (quartiles 55, 70) were included. Prior nephrectomy was performed in 78%. Eastern Cooperative Oncology Group performance status 0–1 was reported in 74%. Lymphatic (68%) and pulmonary (42%) metastases were most common. Of the patients, 59% received first-line immune checkpoint inhibitor (ICI) combination therapies (ICI-ICI: 20%, tyrosine kinase inhibitor [TKI]-ICI: 39%), and 41% of patients received TKI monotherapy, predominantly sunitinib. The median follow-up time was 33.3 mo (interquartile range 14.8–46.7). The median PFS was 5.4 mo (95% confidence interval [CI]: 3.2–7.6) for ICI-ICI combinations, 16.9 mo (95% CI: 7.2–26.6) for ICI-TKI combinations, and 8.8 mo (95% CI: 7.0–10.7) for TKI monotherapy. Of all the patients, 70% and 35% experienced all-grade and grade 3–5 AEs, respectively. AEs of any cause led to discontinuation in 33% of patients.

**Conclusions and clinical implications:**

TKI-based therapies are applied frequently in pRCC patients. Our data support the use of ICI plus TKI as a first-line standard for patients with pRCC. The major limitations were the retrospective data capture and short follow-up of our study. Additional analyses to tailor treatment strategies in patients with metastatic pRCC are warranted.

**Patient summary:**

In this report, we looked at the outcome of first-line treatment of patients with metastatic papillary renal cell cancer (pRCC). Tyrosine kinase inhibitor (TKI)-based therapies are applied frequently in pRCC. Our data support the use of immune checkpoint inhibitor plus TKI as a first-line standard for patients with pRCC. However, further studies are needed to optimise treatment in patients with metastatic pRCC.

## Take Home Message

Our real-word data support the use of a combination of immune checkpoint inhibitors and tyrosine kinase inhibitors in the first-line treatment of papillary renal cell carcinoma. However, prospective trials are needed to establish multimodal therapy in the future and further improve clinical outcomes for our patients.

## 1 Introduction

Renal cell carcinoma (RCC) is one of the more common types of cancer and accounts for 4% of new diagnoses worldwide, with an increasing incidence [1,2]. Histologically, clear cell renal cell carcinoma (ccRCC) dominates in about 80% of cases. Papillary renal cell carcinoma (pRCC) occurs in around 15% of patients with RCC [3]. In 2014, the World Health Organization (WHO) classified pRCC into three subtypes: type 1, which is associated with small tumours with a favourable prognosis and few metastatic development; type 2, commonly more aggressive and associated with a dismal prognosis; and unclassified pRCC [4]. Pathological type 1 is associated with alterations in the MET pathway. Whereas type 2 is associated with the activation of the NRF2-ARE pathway [5]. However, the WHO 2022 classification eliminated type 1/2 pRCC subcategorisation, given the recognition of mixed phenotypes and different molecular backgrounds within the type 2 pRCC category [6].

Tyrosine kinase inhibitors (TKIs) targeting the vascular endothelial growth factor receptor and immune checkpoint inhibitors (ICIs) have become an important part of the systemic treatment of advanced or metastatic renal cell carcinoma (mRCC). Recently, ICI-based combinations have been developed, which have significantly changed the medical treatment of mRCC. There are currently five different options available for first-line therapy [[7], [8], [9], [10], [11]]. However, all randomised phase 3 trials investigating these treatment options are limited to ccRCC subtypes, and only the subgroup of non-ccRCC (nccRCC) is differentiated, which however is composed of several subtypes, with different prognosis and systemic treatment sensitivity [12]. Interestingly, the therapeutic landscape of metastatic pRCC appears to be similar to that of metastatic ccRCC. Small phase 2 trials have shown promising results for ICIs alone or the combination of TKIs and ICIs [13,14]. However, the optimal treatment of metastatic pRCC is still unclear [15].

The broad spectrum of medical treatment options allows for individualised therapy selection to determine the most appropriate treatment option for the patient. Data on this from real patient cohorts are rare. In particular, there is still a lack of data showing a comparison of the different groups of active substances. In this multicentre study, we evaluated the safety and effectiveness of first-line treatment in patients with pRCC in real-world cohorts from 13 centres in Germany.

## 2 Patients and methods

### 2.1 Patient cohort and treatment

Clinical data were collected from 13 oncological centres (in alphabetical order: Cologne, Düsseldorf, Essen, Hannover, Heidelberg, Herne, Jena, Lübeck, Magdeburg, Munich, Münster, Regensburg, and Ulm). Data were retrospectively retrieved from medical records. Patients with advanced or metastatic pRCC who were treated with ICI-ICI or ICI-TKI combination therapy, or TKI monotherapy in the first line were eligible. Patients have received first-line therapy according to local standards from October 2007 to February 2024. Dose adjustments were made at the discretion of the investigators.

Throughout the duration of this study, the ethical standards of the institutional and/or national research committee and the 1964 Declaration of Helsinki, as subsequently amended, were adhered to. The study was approved by the local Ethics Committee of the Medical Faculty of the University Cologne (23-1104-retro).

### 2.2 Statistical analysis

The pseudonymised data sets were analysed with SPSS Statistics 29.0 (SPSS, Armonk, NY, USA). The primary outcome measures were the best overall response rate (ORR) and progression-free survival (PFS). PFS was defined as the time from the start of therapy to the occurrence of radiological or clinical progression or death. The ORR was evaluated by the investigators according to the Response Evaluation Criteria in Solid Tumors version 1.1. Patients were censored at the time of last follow-up if patients were still alive or with unknown death status. AEs were defined according to the Common Terminology Criteria for Adverse Events, version 5.0. The Kaplan-Meier method was used for survival analyses. The duration of the follow-up period was calculated from the time of treatment initiation until death or censoring at the last known follow-up. Prognostic parameters were assessed by a multivariate analysis (Cox regression). Overall, two-sided *p* values of ≤0.05 were considered statistically significant.

## 3 Results

### 3.1 Patient characteristics

A total of 121 patients with pRCC who received systemic first-line treatment were identified. Baseline characteristics are displayed in Table 1. The median age at the initiation of first-line therapy was 63 yr (quartiles 55, 70). Of the patients, 81.8% were <75 yr old at the start of treatment and 77% were male. Eastern Cooperative Oncology Group performance status was 0–1, 2–4, and unknown in 74% (n = 90), 6% (n = 7), and 21% (n = 25) respectively. The International mRCC Database Consortium risk was favourable, intermediate, poor, or unknown in 17% (*n* = 21), 31% (*n* = 38), 23% (*n* = 28), and 28% (*n* = 34) of patients, respectively. Dominant sites of metastases were lymph nodes (68%), lung (42%), bone (28%), and liver (20%). Of the patients, 59% received first-line ICI combinations (ICI-ICI: 20%, TKI-ICI: 39%) and 41% received TKI monotherapy, predominantly sunitinib.

**Table 1:** Characteristics of patients

	All patients (*N* = 121)	ICI-ICI (*n* = 24)	ICI-TKI (*n* = 47)	TKI mono (*n* = 50)
Age at start of treatment (yr), median (quartiles)	63 (55, 70)	63 (54, 72)	63 (58, 69)	61.5 (51, 71)
Age <75 yr, *n* (%)	99 (82)	19 (79)	40 (85)	40 (80)
Gender, *n* (%)				
Male	93 (77)	20 (83)	35 (74)	38 (76)
Female	28 (23)	4 (17)	12 (26)	12 (24)
Prior definitive treatment, *n* (%)				
Yes	94 (78)	15 (63)	34 (72)	45 (90)
T status, *n* (%)				
T1	39 (32)	8 (33)	21 (45)	10 (20)
T2	15 (12)	3 (13)	5 (44)	7 (14)
T3	48 (40)	7 (29)	13 (28)	28 (46)
T4	7 (6)	3 (13)	2 (5)	2 (4)
Unknown	12 (10)	3 (13)	6 (13)	3 (6)
N status, *n* (%)				
N0	34 (28)	3 (13)	15 (32)	16 (32)
N1	35 (29)	9 (38)	12 (26)	15 (30)
Nx	51 (42)	12 (50)	20 (43)	19 (38)
M status, *n* (%)				
M0	19 (16)	3 (13)	7 (15)	9 (18)
M1	102 (84)	21 (88)	40 (85)	41 (82)
ECOG PS, *n* (%)				
0	62 (51)	16 (67)	24 (51)	22 (44)
1	28 (23)	4 (17)	13 (28)	11 (22)
2	5 (4)	1 (4)	3 (6)	1 (2)
3	1 (1)	0 (0)	0 (0)	1 (2)
4	1 (1)	1 (4)	0 (0)	0 (0)
Unknown	25 (21)	2 (8)	8 (17)	15 (30)
IMDC, *n* (%)				
Favourable (0 points)	21 (17)	3 (13)	10 (21)	8 (16)
Intermediate (1–2 points)	38 (31)	5 (21)	17 (36)	16 (32)
Poor (≥3 points)	28 (23)	7 (29)	14 (30)	7 (14)
Unknown	34 (28)	9 (38)	6 (13)	19 (38)
Site of metastases, *n* (%)				
Lymph nodes	82 (68)	19 (79)	27 (57)	36 (72)
Lung	51 (42)	11 (46)	19 (40)	21 (42)
Bone	34 (28)	4 (17)	13 (28)	17 (34)
Liver	24 (20)	6 (25)	7 (15)	11 (22)
Adrenal	7 (6)	2 (8)	5 (11)	0 (0)
Brain	4 (3)	0 (0)	2 (4)	2 (4)
Other	39 (32)	3 (12)	20 (43)	16 (32)
Received second-line therapy, *n* (%)	83 (69)	14 (58)	22 (47)	47 (94)

ECOG PS = Eastern Cooperative Oncology Group performance status; ICI = immune checkpoint inhibitor; IMDC = International mRCC Database Consortium; mono = monotherapy; mRCC = metastatic renal cell carcinoma; TKI = tyrosine kinase inhibitor.

### 3.2 Efficacy of first-line treatment

The median follow-up time from the start of palliative treatment was 33.3 mo (interquartile range 14.8–46.7). At the time of the last follow-up, 68 patients (56%) were alive and 53 (44%) had died. The ORR was documented for 43 and the disease control rate was documented for 78 of all patients. The best response to treatment was a complete response in three patients (Table 2).

**Table 2:** Efficacy of treatment

	All patients(*N* = 121)	ICI-ICI(*n* = 24)	ICI-TKI(*n* = 47)	TKI mono(*n* = 50)
ORR, *n* (%)	43 (36)	8 (33)	19 (40)	16 (32)
CR, *n* (%)	3 (3)	0 (0)	1 (2)	2 (4)
PR, *n* (%)	40 (33)	8 (33)	18 (38)	14 (28)
SD, *n* (%)	35 (29)	4 (17)	16 (34)	15 (30)
PD, *n* (%)	29 (22)	9 (38)	6 (13)	14 (28)
Not evaluable, *n* (%)	14 (12)	3 (13)	6 (13)	5 (10)
DCR, *n* (%)	78 (65)	12 (50)	35 (76)	31 (62)

CR = complete response; DCR = disease control rate; ICI = immune checkpoint inhibitor; mono = monotherapy; ORR = overall response rate; PD = progressive disease; PR = partial response; SD = stable disease; TKI = tyrosine kinase inhibitor.

**Fig. 1:**
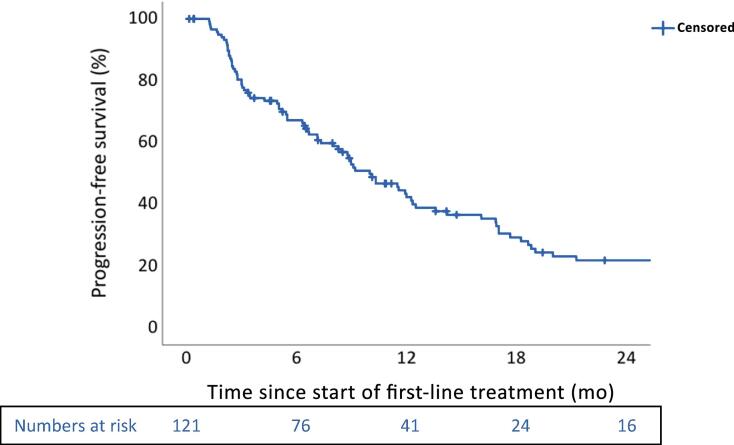
KM plot for PFS of all patients. The median PFS was 10.0 mo (95% CI: 7.7–12.3). CI = confidence interval; KM = Kaplan-Meier; PFS = progression-free survival.

The median PFS (mPFS) was 10.0 mo (95% confidence interval [CI]: 7.7–12.3), and the 6-mo PFS rate was 67.2% (43.3% at 12 mo; Fig. 1).

**Fig. 2:**
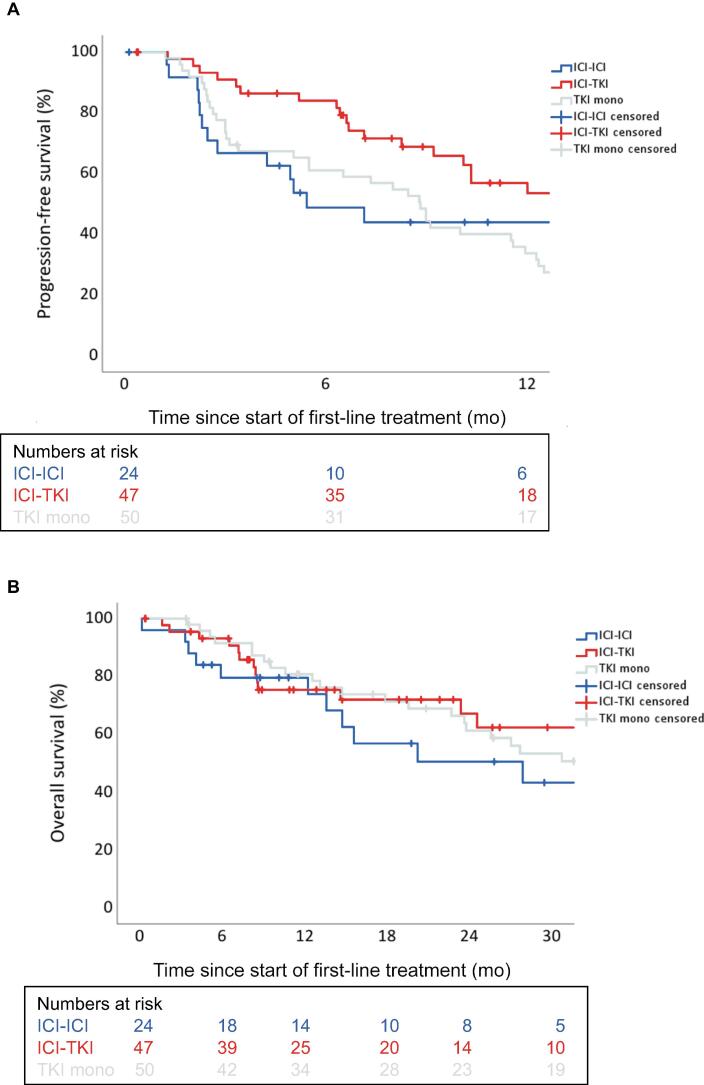
(A) KM plot for PFS in accordance with the type of therapy. The median PFS was 5.4 mo (95% CI: 2.2–8.6) for ICI-ICI combinations, 16.9 mo (95% CI: 7.2–26.6) for ICI-TKI combinations, and 8.8 mo (95% CI: 7.0–10.7) for TKI monotherapy (*p* = 0.01). (B) KM plot for OS in accordance with the type of therapy. The median OS was 27.9 mo (95% CI: 6.5–49.3) for ICI-ICI combinations, not reached for ICI-TKI combinations, and 34.3 mo (95% CI: 21.9–46.6) for TKI monotherapy (*p* = 0.57). CI = confidence interval; ICI = immune checkpoint inhibitor; KM = Kaplan-Meier; mono = monotherapy; OS = overall survival; PFS = progression-free survival; TKI = tyrosine kinase inhibitor.

The 12-mo overall survival (OS) rate was 78.4% for all patients. The median OS (mOS) was 35.4 mo (95 CI%: 25.8–44.9). Next, the effectiveness of the different treatment regimens was investigated. The ORR was achieved in eight, 19, and 16 patients for ICI-ICI combinations, ICI-TKI combinations, and TKI monotherapy, respectively. The mPFS was 5.4 mo (95% CI: 2.2–8.6) for ICI-ICI combinations, 16.9 mo (95% CI: 7.2–26.6) for ICI-TKI combinations, and 8.8 mo (95% CI: 7.0–12.3) for TKI monotherapy (Fig. 2A). The median OS was 27.9 mo (95% CI: 6.5–49.3) for ICI-ICI combinations, not reached for ICI-TKI combinations, and 30.8 mo (95% CI: 19.5–42.0) for TKI monotherapy (Fig. 2B).

**Fig. 3:**
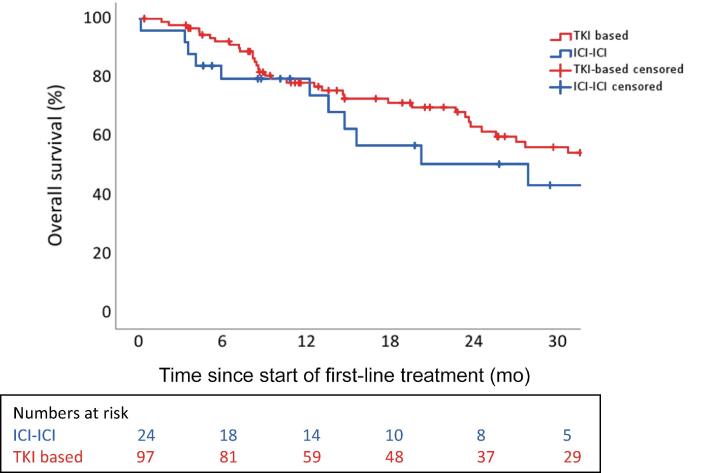
KM plot for OS for ICI-ICI–based versus TKI-based therapy. The median OS was 27.9 mo (95% CI: 6.5–49.3) for ICI-ICI–based therapy and 36.2 mo (95% CI: 24.2–48.2) for TKI-based therapy (*p* = 0.37). CI = confidence interval; ICI = immune checkpoint inhibitor; KM = Kaplan-Meier; OS = overall survival; TKI = tyrosine kinase inhibitor.

There was a significant difference between ICI-ICI and ICI-TKI combination therapies with regard to progressive disease as the best treatment response (9 vs 6, *p* = 0.03). In a direct comparison of the survival analysis between ICI-ICI and ICI-TKI combination therapies, there was a significant difference for mPFS (*p* = 0.02), but not for mOS (*p* = 0.33). A comparison between ICI-ICI–based and TKI-based therapies with regard to mOS also demonstrated a trend in favour of TKI-based therapy, but this was not significant (*p* = 0.37; Fig. 3). A multivariable Cox proportional hazard regression analysis identified improved OS associated with cytoreductive nephrectomy (CN; hazard ratio [HR] 1.9, 95% CI 1.1–3.5, *p* = 0.02) in the overall cohort. In a subgroup analysis, CN was associated with improved OS in patients treated with ICI-TKI–based therapy (HR 3.1, 95% CI: 1.1–8.9), however not in patients treated with ICI-ICI–based therapy (HR 1.0, 95% CI: 0.3–3.6) or TKI mono (HR 1.8, 95% CI: 0.7–4.5).

### 3.3 Safety of first-line treatment

Regarding AEs, 70% of patients experienced any-grade AEs and 35% of patients experienced grade 3–5 AEs. AEs of any cause led to discontinuation in 33% of patients. Next, the AEs of the individual groups of active substances were investigated. AEs of any grade were reported in 75%, 87%, and 52%, and grade 3–5 AEs were reported in 17%, 55%, and 24% for ICI-ICI combinations, ICI-TKI combinations, and TKI monotherapy, respectively. Treatment-related AEs led to discontinuation of treatment in 21%, 45%, and 28% of the ICI-ICI combinations, ICI-TKI combinations, and TKI monotherapy groups, respectively. Regardless of the therapy, the most observed treatment-related AEs were fatigue in 40% (all grades), followed by diarrhoea—25% for all grades. Nausea was noted in 18% of patients. Overall, a grade 5 infection was documented in connection with ICI-TKI combinations.

### 3.4 Second-line treatment

Of all the patients, 83 have received second-line therapy. In second-line treatment, mPFS was 5.5 mo (95% CI: 2.4–8.6) and the PFS rate at 6 mo was 47.5% for all patients. The mPFS was 6.7 mo (95% CI: 3.2–10.3) for patients initially treated with ICI-ICI combinations (mostly TKI monotherapy in second line), 3.4 mo (95% CI: 0.4–6.1) for patients initially treated with ICI-TKI combinations (mostly TKI monotherapy or TKI plus everolimus in second line), and 5.6 mo (95% CI: 0.0–15.5) for patients initially treated with TKI monotherapy (mostly an ICI in second line; Supplementary Table 1).

## 4 Discussion

To our knowledge, this is one of the first retrospective multicentre studies exploring the outcomes of real-world patients with pRCC in the first- and second-line therapy settings. We report data on 121 patients from 13 academic hospitals in Germany.

One of the first prospective studies to investigate the efficacy of sunitinib for pRCC was the SUPAP phase 2 study by the French Genitourinary Group (GETUG). The mPFS was 6.6 mo (95% CI: 2.8–14.8) for type 1 and 5.5 mo (95% CI: 3.8–7.1) for type 2 [16]. The phase 2 AXIPAP trial investigated the efficacy of axitinib as first-line treatment in patients with metastatic pRCC. It showed OS of 18.9 mo (95% CI: 12.8–not reached) [17]. In the phase 2 PAPMET trial, patients were randomised to receive either sunitinib or a MET kinase inhibitor (cabozantinib, crizotinib, or savolitinib). Recruitment was completed for cabozantinib and sunitinib only. PFS was significantly prolonged with cabozantinib compared with sunitinib (9.0 vs 5.6 mo; HR 0.60, 95% CI: 0.37–0.97, *p* = 0.019) [18]. In the phase 2 ASPEN trial, sunitinib improved mPFS (8.1 mo; 80% CI: 5.8–11.4) and mOS (31.5 mo; 95% CI: 14.8–not reached) compared with everolimus. The studies show the heterogeneous results of phase 2 trials, which could be related to the different sizes of the study cohorts and the different histological subtypes of pRCC [19]. However, the mOS for patients in the GUARDIANS cohort with metastatic pRCC treated with a TKI was 30.8 mo (95% CI: 19.5–42.0), and the mPFS was 8.8 mo (95% CI: 7.0–10.7).

ICI-ICI and ICI-TKI combinations in the first-line setting have sustainably improved PFS and OS in metastatic ccRCC [7,[9], [10], [11]], and have thus become new treatment standards [20]. With a follow-up of 96 mo, the CheckMate 214 study reported mPFS of 12.4 mo and mOS of 52.7 mo for the ICI-ICI combination in patients with untreated ccRCC [21]. Nevertheless, the value of an ICI in metastatic nccRCC patients is still debated, as sensitivity appears to vary between subtypes. The nccRCC cohort of the CheckMate 920 study shows a response to ipilimumab and nivolumab in the first-line treatment of patients with metastatic nccRCC. In this study, the following histological subtypes were included: papillary, chromophobe, translocation-associated, collecting duct, medullary, and unclassified. The ORR, mPFS, and OS were 19.6%, 3.7 mo, and 21.2 mo, respectively [22]. The SUNNIFORECAST trial reported an ORR of 32.8%, mPFS of 5.1 mo (95% CI: 2.9–6.1), and mOS of 42.4 mo (95% CI: 35.2–55.5) for patients with pRCC who have been treated with ipilimumab and nivolumab [23]. Our study considers an ORR of 33%, mPFS of 5.4 mo (95% CI: 2.2–8.6), and mOS of 27.9 mo (95% CI: 6.5–49.3) for ICI combination therapy.

The CM9ER study investigated the combination of cabozantinib/nivolumab. After a follow-up of 55.6 mo, PFS was 16.6 mo and mOS was 49.5 mo [24]. In the Keynote-426 study, axitinib/pembrolizumab achieved mPFS of 15.7 mo and mOS of 47.2 mo over the 67-mo follow-up period [25]. For the axitinib/avelumab combination, mPFS of 13.9 mo and mOS of 44.8 mo were observed in the Javelin-Renal-101 study with a follow-up of 72 mo [26]. The CLEAR study demonstrated mPFS of 23.9 mo and mOS of 53.7 mo for lenvantinib/pembrolizumab at a follow-up of 48 mo [27]. All the above-mentioned studies led to the approval in the first-line treatment of mRCC. However, only patients with ccRCC were included here. As far as we are aware, there are no studies to date that have investigated the efficacy of ICI-TKI combinations in an exclusively metastatic pRCC population. In our cohort, most of the patients treated with ICI-TKI combinations received either nivolumab/cabozantinib (*n* = 14) or pembrolizumab/axitinib (*n* = 26). Cabozantinib and nivolumab were evaluated in a phase 2 study in patients with nccRCC. The mOS was 28 mo (95% CI: 16.3–not reached) and mPFS was 12.5 mo (95% CI: 6.3–16.4) [14]. In the KEYNOTE-B61 trial, the effectiveness of pembrolizumab plus lenvantinib for nccRCC was examined. Of all the patients, 54% had pRCC; mPFS was 18 mo (95% CI: 14–not reached) and mOS was not reached for all the patients. Analyses of mPFS among pRCC patients resulted in PFS of 17.5 mo (95% CI: 15–not reached) [28]. One of the few retrospective analyses that included pRCC patients only and examined the effectiveness of ICI-TKI combinations reported mOS of 17.4 mo (95% CI: 10.1–30.0) [29]. In our cohort, mOS was not reached and mPFS was 16.9 mo (95% CI: 7.2–26.6) for ICI-TKI combinations. Moreover, a significantly lower number of patients treated with ICI-TKI combinations showed progressive disease than patients treated with ICI-ICI combinations. A direct comparison of the lifetime analyses between these patient groups has shown significant differences for mPFS but not for mOS, which could be a consequence of the short follow-up. However, our data might ultimately suggest a trend in favour of the combinations of TKIs and ICIs, and support the use of TKIs and ICIs as a first-line standard for patients with pRCC. However, it is still not clear which patient group will benefit from the combination therapy and which could do without immunotherapy.

CN proved to be an independent prognostic factor for OS (HR 1.9, 95% CI 1.1–3.5, *p* = 0.02) in our patient cohort, especially in the IO-TKI cohort. These results are comparable with the data from the study of Luzzago et al [30], in which CN was also associated with an improvement in OS in patients with metastatic pRCC. CN thus appears to have a strong influence on the response to therapy. However, the influence of CN depending on further systemic therapy is currently unclear. Currently on-going trials are investigating the influence of CN on immune-based therapy [31,32].

Molecular changes in the signalling pathways are often detectable in pRCC, which can either be prognostic or have an influence on therapeutic sensitivity. In particular, pRCC is characterised by genomic abnormalities that lead to dysregulation of the MET pathway. In the phase 3 SAVOIR study, although none of the study endpoints reached significance, the limited efficacy data in this study favoured savolitinib over sunitinib [33]. This is in line with the previous results, where patients with MEK alteration benefited from savolitinib [34]. Actually, the phase 3 SAMETA study investigates the efficacy of savolitinib, a MET inhibitor, in combination with durvalumab versus sunitinib and durvalumab monotherapy [35]. Another interesting molecular alteration is mutation in NRF2-ARE, which could be associated with poorer prognosis. Inhibition of NRF2 could represent a further therapeutic approach for MET-independent pRCC patients [36]. In addition, mutations in CDKN2A and CDKN2B were associated with poor clinical outcomes and could be used as biomarkers. For example, sarcomatoid tumours often show CDKN2A/B alterations and increased PD-L1 expression. These findings can be used to explain the better results of sarcomatoid tumours with checkpoint blockade [37].

Limitations of our study are its retrospective nature with selection and recall biases as well as the short follow-up time. We did not utilise a radiological blinded independent central review in our analysis.

## 5 Conclusions

In summary, our real-word data support the use of a combination of ICIs and TKIs in the first-line treatment of pRCC. Furthermore, CN is an independent factor for OS. However, prospective trials are needed to establish multimodal therapy in the future and further improve clinical outcomes for our patients.

  ***Author contributions*:** Thomas Hilser had full access to all the data in the study and takes responsibility for the integrity of the data and the accuracy of the data analysis.

  *Study concept and design*: Hilser, Paffenholz.

*Acquisition of data*: Hilser, Paffenholz, Casuscelli, Aydogdu, Zschäbitz, Schnabel, Rinderknecht, Mattigk, Schostak, Volk, Ivanyi, Wiegmann, Darr, Flegar, Mandal, Schlack, Seidl, Handke, Klee, Nestler, Rehlinghaus, Hijazi, Heidenreich, Grünwald.

*Analysis and interpretation of data*: Hilser, Paffenholz.

*Drafting of the manuscript*: Hilser.

*Critical revision of the manuscript for important intellectual content*: Paffenholz, Casuscelli, Aydogdu, Zschäbitz, Schnabel, Rinderknecht, Mattigk, Schostak, Volk, Ivanyi, Wiegmann, Darr, Flegar, Mandal, Schlack, Seidl, Handke, Klee, Nestler, Rehlinghaus, Hijazi, Heidenreich, Grünwald.

*Statistical analysis*: Hilser.

*Obtaining funding*: None.

*Administrative, technical, or material support*: None.

*Supervision*: Paffenholz.

*Other*: None.

  ***Financial disclosures:*** Thomas Hilser certifies that all conflicts of interest, including specific financial interests and relationships and affiliations relevant to the subject matter or materials discussed in the manuscript (eg, employment/affiliation, grants or funding, consultancies, honoraria, stock ownership or options, expert testimony, royalties, or patents filed, received, or pending), are the following: T. Hilser: honoraria—Ipsen. J. Casuscelli: travel, accommodations, and expenses—Bayer, Janssen Oncology, Merck KGaA, and Pfizer; expert testimony—Janssen-Cilag, MSD Oncology, and Astellas Pharma; research funding—AstraZeneca and MSD Oncology. C. Aydogdu: travel, accommodations, and expenses—Ipsen, Eisai, and Janssen; honoraria—Ipsen and Astellas Pharma. S. Zschäbitz: consulting or advisory role—Pfizer, MSD Oncology, Merck, Sanofi/Aventis, EUSA Pharma, Roche, Bristol-Myers Squibb, Eisai, Amgen, Astellas Pharma, Ipsen, Bayer, and Gilead Sciences; travel, accommodations, and expenses—Merck, Pfizer, BMS GmbH & Co. KG, Ipsen, Amgen, Astellas Pharma, and Janssen Oncology; honoraria—Amgen, Eisai Germany, Merck Serono, Pfizer, MSD Oncology, BMS GmbH & Co. KG, Janssen Oncology, Ipsen, Astellas Pharma, Novartis, and Bayer; research funding—Eisai. M.J. Schnabel: consulting or advisory role—Pfizer, AstraZeneca, Johnson & Johnson/Janssen, Ipsen, Bayer, Novartis, Gilead Sciences, and MSD Oncology; travel, accommodations, and expenses—Ipsen, Pfizer, and Johnson & Johnson/Janssen; stock and other ownership interests—BioNTech; honoraria—Bayer, Ipsen, Pfizer, Johnson & Johnson/Janssen, Astellas Pharma, AstraZeneca, Gilead Sciences, Merck Serono, MSD Oncology, Medac, Apogepha, Novartis, AstraZeneca, Ipsen, and MSD Oncology. E. Rinderknecht: honoraria—Ipsen. P. Ivanyi: consulting or advisory role—Bayer/Vital, Bristol-Myers Squibb, ClinSol, Deciphera, Eisai, EUSA Pharma, Ipsen, Merck Serono, MSD, and Pfizer; travel, accommodations, and expenses—Bristol-Myers Squibb, Ipsen, and Merck Serono; stock and other ownership interests—BB Biotech Ventures; honoraria—AIMM Therapeutics, AstraZeneca, Bayer/Vital, Bristol-Myers Squibb, Eisai, EUSA Pharma, Ipsen, Merck Serono, MSD Oncology, and Roche Pharma AG. C. Darr: consulting or advisory role—Bayer, Ipsen, and Janssen; travel, accommodations, and expenses—Bayer, Ipsen, Janssen, and Xeos Medical; stock and other ownership interests—Bayer; honoraria—Bayer, Ipsen, Janssen, and MSD. K. Schlack: consulting or advisory role—Apogepha, Bayer, BMS GmbH & Co. KG, Ipsen, Janssen, and Pfizer; travel, accommodations, and expenses—AstraZeneca, Bayer, Ipsen, Janssen, and Pfizer; honoraria—AAA HealthCare, Amgen, Astellas Pharma, AstraZeneca, Bayer Germany, Eisai Germany, Fosanis, Ipsen, Janssen, Merck Serono, MSD, Novartis, and Pfizer. T. Nestler: honoraria—Janssen. M. Rehlinghaus: consulting or advisory role—AstraZeneca, Merck, Ipsen, Bayer, Novartis, and Johnson & Johnson; honoraria—Apogepha, Merck, and AstraZeneca. A. Heidenreich: consulting or advisory role—Astellas Pharma, AstraZeneca, Bayer, BMS Global, Clovis Oncology, Janssen-Cilag, and MSD Oncology; honoraria—Amgen, Astellas Pharma, Bayer, Ferring, Ipsen, Janssen-Cilag, Sanofi, and Takeda; speakers' bureau—Amgen, Astellas Pharma, Bayer, Ipsen, Johnson & Johnson, Pfizer, Sanofi, and Takeda; research funding—BMS Oncology. V. Grünwald: consulting or advisory role—Bristol-Myers Squibb, Cureteq, Debiopharm Group, Eisai, Gilead Sciences, Ipsen, Janssen-Cilag, MSD Oncology, Novartis, Oncorena, PCI Biotech, Pfizer, and Synthekine; travel, accommodations, and expenses—AstraZeneca, Ipsen, Janssen, Merck Serono, and Pfizer; stock and other ownership interests—AstraZeneca, Bicycle Therapeutics, Bristol-Myers Squibb, Genmab, and MSD; honoraria—Novartis, Amgen, Apogepha, Astellas Pharma, AstraZeneca, Bristol-Myers Squibb, Eisai, Ipsen, Janssen-Cilag, Merck Serono, MSD Oncology, Ono Pharmaceutical, and Pfizer; research funding—Amgen, Bicycle Therapeutics, BMS, Gilead Sciences, Ipsen, MSD Oncology, and Seagen. P. Paffenholz: consulting or advisory role—AstraZeneca, BMS GmbH & Co. KG, Ipsen, Janssen, Merck, MSD, Novartis, Pfizer, and Roche; travel, accommodations, and expenses—Astellas Pharma, AstraZeneca, Ipsen, Janssen, Medac, and Merck; honoraria—Apogepha, Astellas Pharma, AstraZeneca, BMS GmbH & Co. KG, Eisai, Ipsen, Janssen, Merck, and Novartis. The remaining authors declare no conflict of interest.

  ***Funding/Support and role of the sponsor*:** We acknowledge support by the Open Access Publication Fund of the University of Duisburg-Essen.

  ***Ethics statement*:** This retrospective analysis was approved by the Ethics Committee of the Medical Faculty of the University Cologne (23-1104-retro). Written informed consent for participation was not required for this study in accordance with the national legislation and the institutional requirements.

  ***Data sharing statement*:** The datasets generated for this study are available on request to the corresponding author.
