# Supplementary material for: Real-world Evidence from a Retrospective Multicentre Analysis on First-line Therapy for Metastatic Papillary Renal Cell Carcinoma. A GUARDIANS Project
Source: Eur Urol Open Sci. 2025 Jul 12;78:59–67. doi: 10.1016/j.euros.2025.06.011 (PMC12280342; doi:10.1016/j.euros.2025.06.011)
Supplement: Supplementary Data 1 [file mmc1.docx]

**Supplementary Table 1.** Efficacy of 2^nd^ line treatment

|  | All patients  (N=121) | ICI-ICI  (N = 24) | ICI-TKI  (n =47) | TKI mono  (n=50) |
| --- | --- | --- | --- | --- |
| 2^nd^ line treatment, n (%) | 83 (69) | 14 (58) | 22 (47) | 47 (94) |
| ICI in  2^nd^ line treatment, n (%) | 26 (22) | 0 (0) | 2 (4) | 24 (48) |
| TKI mono  in 2^nd^ line treatment , n (%) | 38 (31) | 12 (50) | 14 (30) | 12 (24) |
| TKI plus Everolimus  in 2^nd^ line treatment, n (%) | 8 (7) | 1 (4) | 6 (13) | 1 (2) |
| Everolimus  In 2nd line treatment, n (%) | 6 (5) | 0 (0) | 0 (0) | 6 (12) |
| Other therapies  in 2^nd^ line treatment, n (%) | 5 (4) | 1 (4) | 0 (0) | 4 (8) |
| mPFS (95%-CI) | 5.5 (2.4-8.6) | 6.7 (3.2-10.3) | 3.4 (0.4-6.1) | 5.6 (0.0-15.5) |
